# Hepatic Artery Embolization prior to En Bloc Resection of an Encased Common Hepatic Artery in Adenocarcinoma of the Head of the Pancreas

**DOI:** 10.1155/2013/205475

**Published:** 2013-08-04

**Authors:** Gregory Sergeant, Erik Schadde, Geert Maleux, Raymond Aerts

**Affiliations:** ^1^Department of Abdominal Surgery, University Hospital Leuven, Herestraat 49, 3000 Leuven, Belgium; ^2^Swiss HPB Center, Department for Visceral and Transplantation Surgery, University Hospital Zurich, 8091 Zurich, Switzerland; ^3^Department of Radiology, University Hospital Leuven, Herestraat 49, 3000 Leuven, Belgium

## Abstract

A 64-year-old female patient with adenocarcinoma of the head of the pancreas with encasement of the common hepatic artery and portal vein stenosis was reexplored after six cycles of gemcitabine (1000 mg/m^2^). Prior to surgery, the patient underwent balloon dilation and stenting of the portal vein in addition to successful coil embolisation of the common hepatic artery, proper hepatic artery, and proximal gastroduodenal artery. After embolisation, a pylorus-preserving pancreaticoduodenectomy was performed with resection of the common hepatic artery and portal vein confluens. Pathological examination showed a moderately differentiated pT3N0 (Stage IIa, TNM 7th edition) tumor with negative section margins. We show with this case that in selected cases of periampullary cancer with encasement of the common hepatic artery, it is technically feasible to perform pancreaticoduodenectomy with hepatic artery resection and negative surgical margins. Nevertheless, the oncological benefit of extended arterial resections remains controversial.

## 1. Introduction

Arterial invasion in adenocarcinoma of the head of the pancreas generally precludes curative pancreaticoduodenectomy. Certainly, preservation of good hepatic arterial flow is important to avoid hepatobiliary complications such as liver necrosis, liver abscesses, or bilioenteric anastomotic complications. In selected cases, an R0 pancreaticoduodenectomy with complex hepatic and/or superior mesenteric artery reconstruction is feasible with acceptable perioperative morbidity and postoperative survival up to 15 months [1].

Also, in carcinoma of the body or tail of the pancreas with invasion of the celiac axis, extended pancreatectomy with en bloc celiac axis resection (i.e., Appleby's procedure) is feasible and may prolong survival as opposed to those patients who undergo palliative therapy. Some centers have gained some experience in preoperative embolization of a tumor-encased replaced right hepatic artery to allow for sufficient collateralization prior to pancreaticoduodenectomy with arterial resection [2]. To our knowledge, preoperative coil embolization of the entire arterial tree including common hepatic artery, proper hepatic artery, and proximal gastroduodenal artery prior to pancreaticoduodenectomy for pancreatic adenocarcinoma in order to obtain negative section margins has not been previously reported. We report a case where this was attempted.

## 2. Methods

**Figure 1:**
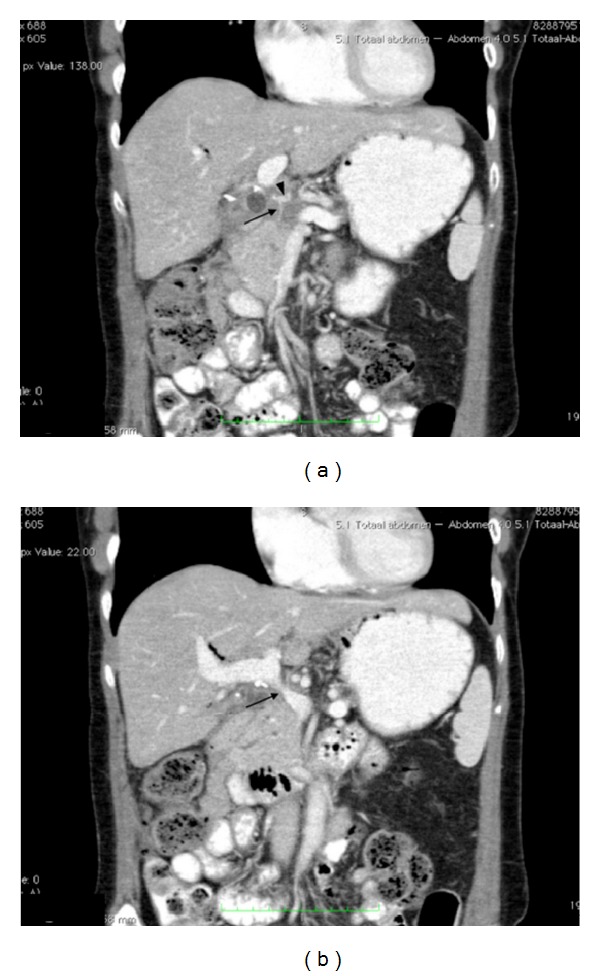
(a) 3 months interval portal venous-phase abdominal CT scan (coronal view). Status after exploratory laparotomy and 6 courses of chemotherapy. Note hypodensity in pancreatic head with encasement of the gastroduodenal artery (arrow) and common hepatic artery (arrowhead). (b) 3 months interval portal venous-phase abdominal CT scan (coronal view). Status after exploratory laparotomy and 6 courses of chemotherapy. Progressive stenosis of the portal vein can be noted (arrow).

A 64-year-old female patient with pancreatic ductal adenocarcinoma (PDAC) staged uT3N1 and abutment of the common hepatic artery underwent surgical exploration and was declared unresectable because of true encasement of the common hepatic artery at the origin of the gastroduodenal artery. A palliative hepaticojejunostomy was fashioned. After six courses of gemcitabine (1000 mg/m^2^) stable disease according to RECIST criteria (version 1.1) was noted on consecutive CT scan. Nevertheless, during chemotherapy, the portal vein became progressively stenotic over a short segment with subsequent venous collateralization (Figures 1(a) and 1(b)). Arterial collateralisation secondary to the encasement was not present. Also, CA19.9 decreased to normal range during chemotherapy.

### 2.1. Interventional Radiology

**Figure 2:**
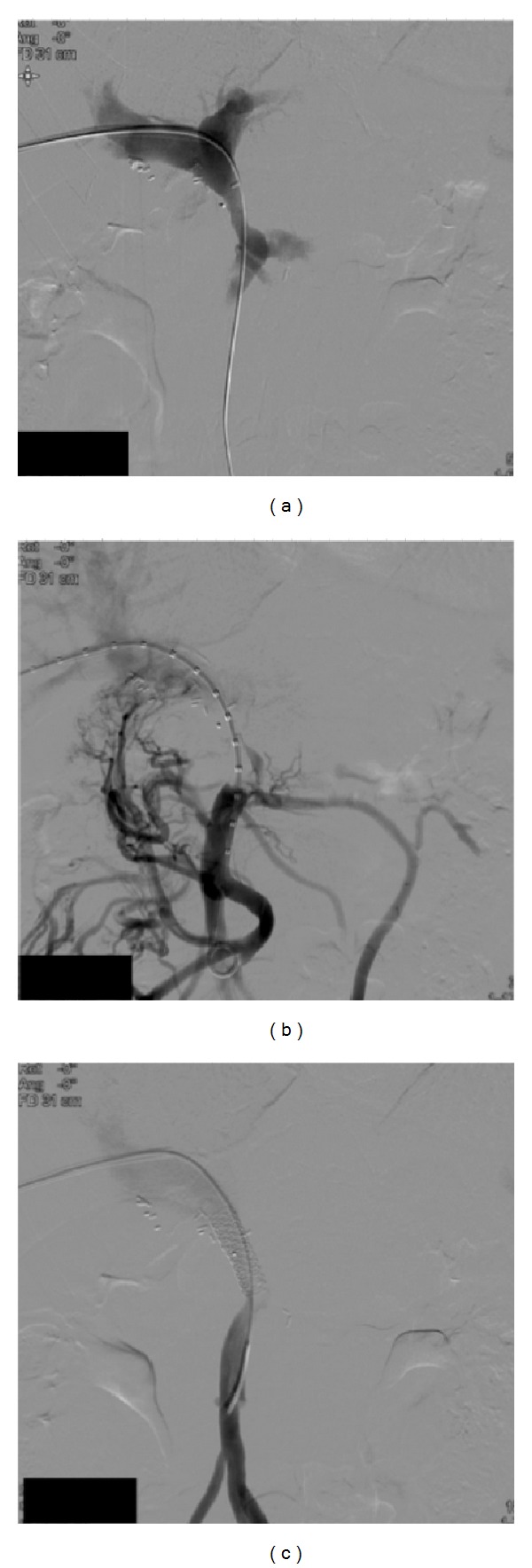
(a) Percutaneous portography. A segmental stenosis is noted cranial to the portal vein—splenic vein junction. (b) Percutaneous portography. As a consequence of the portal vein stenosis, extensive collateralisation takes place. (c) Percutaneous portography. After balloon dilation, a stent is inserted. Angiographic control confirms the improved lumen diameter and immediate loss of compensatory collateral flow.

**Figure 3:**
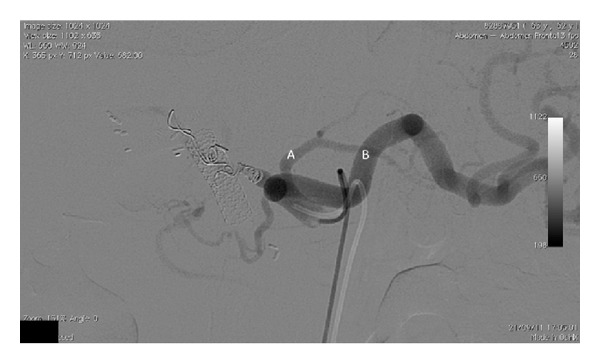
Arteriography of the celiac trunk after successful embolisation of the common hepatic artery and gastroduodenal artery. The left gastric artery (A) and splenic artery (B) remain patent.

Under general anesthesia, percutaneous balloon dilation (6 × 20 mm and 9 × 40 mm) and stenting (Scuba 9 × 30 mm, Invatec, Roncadelle, Italy) of the portal vein were performed after ultrasound-guided catheterization of the right portal vein (Figures 2(a)–2(c)). In addition, coil embolisation of the common hepatic artery, proper hepatic artery, and proximal gastroduodenal artery (2 × Nester 18/7/6, 1 × Nester 18/7/4 and 5 × Tornado 18s - 3/2, Cook Medical, Bjaeverskov, Denmark) was performed via catheterization of the right femoral artery (Figure 3). The right and left hepatic arteries are originated from the proper hepatic artery. 

### 2.2. Surgical Technique

Two weeks after embolization, an extended pylorus-preserving pancreaticoduodenectomy was performed with en bloc resection of the common hepatic artery and portal vein-splenic vein confluens and para-aortic lymphadenectomy. The portal vein stent was removed during the venous resection. The existing hepaticojejunostomy was taken down to permit the pancreatectomy. The portal vein-superior mesenteric vein continuity was restored with an end-to-end anastomosis using prolene 5/0. The splenic vein was suture-ligated. A pancreaticogastrostomy was fashioned in two layers using PDS 4/0. An end-to-side hepaticojejunostomy was reconstructed with interrupted sutures PDS 5/0. Finally, an antecolic pylorojejunostomy was constructed in two layers. Total operating time was 375 minutes. Total blood loss was estimated at 4000 mL. Intraoperative transfusion consisted of 7 units of erythrocyte concentrate and 4 units of fresh frozen plasma. 

## 3. Results

The postoperative course was complicated (Clavien-Dindo grade II) with delayed gastric emptying, necessitating prolonged nasogastric tube and prokinetics, a superficial surgical site infection and fever with positive drainage fluid cultures necessitating intravenous piperacillin/tazobactam for 7 days. Postoperative liver transaminases AST and ALT increased to a maximum of 4809 and 5444 IU/L on postoperative day 1 and returned to normal by postoperative day 9. The patient was discharged on postoperative day 20.

Pathological examination showed a moderately differentiated pT3N0 (Stage IIa, TNM 7th edition) pancreatic ductal adenocarcinoma with a maximal diameter of 45 mm.

All section margins were negative. Hepatic artery invasion was absent. Despite extensive lymphadenectomy, only four negative nodes were found. Perineural and lymphovascular invasions were present. 

The patient was readmitted 1.5 months after surgery because of a decline in her general condition, nausea, anorexia, and a swollen abdomen. Imaging revealed a local recurrence with malignant ascites and invasion of the liver hilum. Palliative gemcitabine was initiated; however, the patient's condition worsened and she finally died 83 days after surgery.

## 4. Discussion

Hepatic artery resection followed by reconstruction is uncommon as it requires microvascular reconstruction techniques and the incidence of complications is high. Selecting patients for an aggressive strategy with arterial resection is difficult. Patients with favorable response to preoperative therapy (radiographical evidence of tumor regression and improvement in serum tumor marker levels) are the subset of patients who have the best chance for an R0 resection and a favorable long-term outcome [3]. Nevertheless, our patient developed early local recurrence with rapid mortality. A clear reason for this has not been demonstrated, although one may argue that preoperative chemotherapy with gemcitabine alone is suboptimal. On the other hand, the benefit of neoadjuvant chemotherapy or chemoradiotherapy in this setting is still not clear. Indeed, chemoradiotherapy may result in downsizing of the tumor and subsequent better locoregional control; however, its beneficial effect on the overall survival remains to be confirmed. 

In order to avoid some of the morbidity associated with arterial resection and reconstruction, we propose a strategy of embolization of the hepatic artery in a case of incidental downstaging. In addition, in order to maintain good perfusion of the liver after embolization, the stenosed portal vein was stented prior to arterial embolization. To our knowledge, this approach has not been published yet. In addition, portal vein stenting may reduce venous collateralization and subsequent intraoperative blood loss, a strategy that has been used successfully prior to liver transplantation in the setting of portal vein thrombosis [4].

Patients undergoing pancreatic resection without additional risk factors have a risk of liver ischemia of 2.2% [5]. Simultaneous involvement of the portal vein and hepatic artery was always fatal. Preoperative embolization of the common hepatic artery has been proposed by some groups to decrease ischemia-related events, such as gallbladder and hepatic necrosis in cases of pancreatic cancer of the body or tail requiring a celiac trunk resection without reconstruction [6]. The development of vascular collaterals to the liver following embolization of the CHA, PHA, and origin of the GDA occurs via the inferior phrenic artery, but also or even more so via the superior mesenteric artery via the retroperitoneal pancreaticoduodenal arcades to the gastroduodenal and main hepatic arteries [7]. In most patients, collateralization is already noted within 1 or 2 weeks after embolization and subsequent angiograms show progressive enlargement. Plengvanit et al. also showed that after collateralization, the branches of the hepatic artery fill up to the occluded portions via a retrograde fashion, therefore, allowing sufficient arterial blood flow to reach the bile duct stump [8]. In our patient, increased collateralization via the superior mesenteric artery resulted in a substantial increase in the blood loss (i.e., 4000 mL) necessitating blood transfusion during pancreaticoduodenectomy. Major blood loss may substantially increase the perioperative morbidity and mortality and therefore remains a major downside of our technique. However, despite the immediate postoperative rise in liver transaminases, no other liver-related complications occurred.

In conclusion, we believe that in pancreatic ductal adenocarcinoma with encasement of the common hepatic artery, it is technically feasible to perform pancreaticoduodenectomy with hepatic artery resection and negative surgical margins. However, in spite of negative margins, long-term oncological outcome is still compromised. Therefore, we only recommend this strategy in highly selected patients with a strong drive for an aggressive surgical management.
